# MiR-7-5p suppresses tumor metastasis of non-small cell lung cancer by targeting NOVA2

**DOI:** 10.1186/s11658-019-0188-3

**Published:** 2019-11-20

**Authors:** Haiping Xiao

**Affiliations:** Thoracic Surgery Department, General Hospital of Southern Theater Command, Guangzhou, 510010 PLA China

**Keywords:** Non-small cell lung cancer (NSCLC), microRNA-7-5p (miR-7-5p), Suppress, Metastasis, Neuro-oncological ventral antigen 2 (NOVA2)

## Abstract

**Background:**

Non-small cell lung cancer (NSCLC) is the leading cause of cancer mortality worldwide. Distant metastasis is thought to be one of the most important factors responsible for the failure of NSCLC therapy. MicroRNA-7-5p (miR-7-5p) has been demonstrated to be a tumor suppressor in breast cancer, hepatocarcinoma, prostate cancer and glioblastoma multiforme (GBM). However, its role in NSCLC is still not fully understood. This study evaluated the role of miR-7-5p in the progression of NSCLC and explored the underlying mechanism.

**Materials & methods:**

The quantitative real-time PCR (qPCR), MTT, migration and invasion assays were used to evaluate the effects of miR-7-5p on the proliferation, migration and invasion of A549 and SPCA-1 cells. A tumor xenograft model was created to determine the effects of miR-7-5p on metastasis in vivo. The dual-luciferase reporter gene, neuro-oncological ventral antigen 2 (NOVA2) overexpression and western blotting assays were performed to explore the underlying mechanism.

**Results:**

MiR-7-5p is downregulated in NSCLC tissues and lung cancer cell lines. It suppresses proliferation, migration, invasion and EMT marker expression in vitro and in vivo. Further study showed that miR-7-5p suppresses tumor metastasis of NSCLC by targeting NOVA2. Overexpression of NOVA2 attenuates the miR-7-5p-mediated inhibitory effect on lung cancer cells.

**Conclusion:**

MiR-7-5p suppresses NSCLC metastasis. Targeting miR-7-5p may contribute to the success of NSCLC therapy.

## Introduction

Lung cancer is not only one of the most common malignancies, it also has the highest morbidity and mortality of any cancer. More than 85% of all lung cancer cases are non-small cell lung cancer (NSCLC) [1, 2]. Although advances have been made in preclinical and clinical trials for NSCLC therapy, the results are still unsatisfactory, with only 15% of patients living 5 years after diagnosis [3].

Distant metastasis is thought to be one of most important factors responsible for the failure of NSCLC therapy. The mechanism of metastasis has not yet been fully explored. Identifying key molecules involved in NSCLC metastasis is crucial for new and effective anti-NSCLC therapy [1, 4].

MicroRNAs (miRNAs) are a category of highly conserved, endogenously expressed small noncoding RNA. They function as major players in post-transcriptional gene expression through direct interaction with the 3′-untranslated region (3′-UTR) of corresponding target messenger RNAs (mRNAs) and through miRNA cleavage [5, 6]. Multiple studies have shown that miRNAs can be tumor promoters or suppressors. Many also play key roles in metastasis of certain cancers, including gastric cancer, breast cancer, hepatocellular carcinoma, bladder cancer and NSCLC. For example, miR-200 is downregulated in cancer cells with highly metastatic abilities and its overexpression reverses epithelial-mesenchymal transition (EMT) phenotype. MiR-135b has been demonstrated to promote lung cancer metastasis [7–9]. It is also noteworthy that miRNAs may be useful for early diagnosis and therapy or as predictive factors for patient survival and prognosis [10, 11].

MicroRNA-7 (miR-7) is a fascinating miRNA that plays diverse roles in physiological and pathological conditions. In humans, miR-7 is transcribed from miR-7-1, miR-7-2 and miR-7-3, both of which have the same mature miRNA sequence. MicroRNA-7-5p (miR-7-5p) is the most investigated miRNA sequence in this family [12, 13].

Many studies have demonstrated that miR-7-5p is a tumor suppressor in breast cancer, hepatocarcinoma, prostate cancer and glioblastoma multiforme (GBM). Several recent studies showed that miR-7-5p plays a vital role in tumor metastasis. It inhibits the proliferation, migration and invasion abilities of tumor cells through direct targeting of PI3K/Akt, FAK and KLF4 expression. It can also inhibit the metastasis of melanoma cells by regulating RelA/NF-κB [13–17]. However, the involvement of miR-7-5p in NSCLC metastasis and the underlying mechanism remain to be elucidated.

NOVA2 is a member of the Nova family of neuron-specific RNA-binding proteins. NOVA2 and NOVA1, two of the most important subtypes, play critical roles in the survival and proper development of motor neurons [18, 19]. Recently, NOVA1 has been in focus for its contributions to the metastasis and development of astrocytoma, gastric carcinogenesis and lung cancer [20–22]. However, the function of NOVA2 in tumor development is poorly understood.

In this study, we determined the miR-7-5p mRNA level in NSCLC tissues and adjacent normal lung tissues. We showed that miR-7-5p expression decreases in NSCLC tissues and cell lines, and its low expression suggests a poor prognosis for NSCLC patients. Further study showed that miR-7-5p suppresses tumor metastasis of NSCLC by targeting neuro-oncological ventral antigen 2 (NOVA2). We also found that miR-7-5p suppresses tumor growth and metastasis in A549 xenografts.

## Materials and methods

### Materials

Dulbecco’s modified Eagle medium (DMEM), fetal bovine serum (FBS), penicillin–streptomycin (PS) and Lipofectamine LTX & PLUS reagents were obtained from Thermo Fisher Scientific. The Dual-Luciferase Reporter Assay System was purchased from Promega. Transwell plates (6.5 mm) with 8.0-μm pore polycarbonate membrane inserts and other cell culture consumables were purchased from Corning. Matrigel was purchased from BD Biosciences. The pCMV3-NOVA2-GFPSpark and pCMV3-C-GFPSpark vectors were obtained from Sino Biological. The RNA Extraction Kit was purchased from Omega Bio-Tek. MiR-7-5p mimic (5′-UGGAAGACUAGUGAUUUUGUUGU-3′) and NC mimic (5′-UUUGUACUACACAAAAGUACUG-3′) were synthesized by RiboBio. The NC and NOVA2 vectors were purchased from Origene. Antibodies against NOVA2 were purchased from Abcam. Antibodies against vimentin, snail, slug, ZEB1, N-cadherin, E-cadherin, ZO-1 and β-tubulin were purchased from Cell Signaling Technology. Other reagents were purchased from Sigma-Aldrich.

### Patients and tissue samples

50 pairs of NSCLC and adjacent normal lung tissue samples were collected from patients at the General Hospital of Southern Theater Command, PLA, Guangzhou, China. None of the patients had received any radio- or chemotherapy before surgery. The study was approved by the Ethics Committee of General Hospital of the Southern Theater Command (approval number: LL-KT-2018-120). All patients gave written informed consent. All tissues were verified by two independent pathologists. The samples were snap-frozen immediately after resection and stored in liquid nitrogen.

### Cell lines

The human lung cancer cell lines A549 (ATCC CCL-185), NCI-H358 (ATCC CRL-5807) and NCI-H460 (ATCC HTB-177), and human lung epithelial cell line BEAS-2B (ATCC CRL-9609) were purchased from the American Type Culture Collection (ATCC). Human lung cancer cell lines SPC-A-1 (CCTCC NO 500 GDC063) and XL-2 (CCTCC NO C201282) were from the China Center for Type Culture Collection (CCTCC). All cells were maintained in DMEM supplemented with 10% FBS (Biowest) at 37 °C in an incubator with a humidified atmosphere containing 5% CO_2_.

### Animals

Male BABL/c (nu/nu) mice were obtained from Vital River Laboratory Animal Technology and maintained in a specific pathogen-free room that with free access to water and standard laboratory chow. The animal experiments were approved by the Laboratory Animal Ethics Committee of General Hospital of Southern Theater Command in accordance with the ARRIVE guidelines (approval number: 20180824114354).

### Tissue sample preparation and RNA isolation

Total RNA of the frozen tissues were isolated with a TRIzol reagent following the manufacturer’s instructions (Invitrogen). The concentration and quality of RNA spectrophotometrically were determined by measuring the optical density (A260/280 > 2.0; A260/230 > 1.8) with a Nanodrop ND-1000 (Thermo Fisher Scientific).

### Quantitative reverse-transcription PCR (qRT-PCR)

After extracting total RNA, a Transcriptor First Strand cDNA Synthesis Kit (Takara) and All-in-One miRNA qRT-PCR detection kit (GeneCopoeia) were respectively used to generate cDNA from mRNA and miRNA following the manufacturer’s protocol. The oligonucleotide primers used to detect miR-7-5p and NOVA2 were: miR-7-5p primers (5′-GCGCTGGAAGACTAGTGATTTTGTTGTT-3′), NOVA2 primers (forward 5′-GGGTTCCCATAGACCTGGAC-3′, reverse 5′-CGCTCAGTAGTACCTGGGTAA-3′), GAPDH (a housekeeping gene for mRNA) primers (forward 5′-GTGAACCATGAGAAGTATG-3′, reverse 5′-CGGCCATCACGCCACAGTTTC-3′) and U6 (a housekeeping gene for miRNA) primers (5′-CTCGCTTCGGCAGCACA-3′). The universal reverse primer was (5′-AACGCTTCACGAATTTGCGT-3′). The primers, cDNA and SYBR Green I Master Mix were mixed to form the PCR system. The PCR conditions were: 45 cycles of 95 °C for 10 s, 60 °C for 20 s and 72 °C for 20 s, and quantitative real-time PCR was performed with a Roche Lightcycler 480 Real-Time PCR machine. All experiments were repeated three times with different samples in each group. The value of the housekeeping gene was set as 1, and the target gene levels are presented as the fold change relative to the housekeeping gene.

### Cell proliferation assay

The cell viabilities were evualgted with 3-(4, 5-dimethylthiazol-2-yl)-2, 5-diphenyltetrazolium bromide (MTT) assay as previously described [23]. Briefly, a certain number of A549 and SPCA-1 cells (1 × 10^4^ cells per well for the cell viabilities at 24 h, 0.7 × 10^4^ for 48 h, 0.5 × 10^4^ for 72 h and 0.3 × 10^4^ for 96 h) were seeded in 96-well plates and cultured for 24 h. The adherent cells were incubated with or without miR-7-5p for 24, 48, 72 or 96 h. After that, the cell viability was determined using the MTT assay. Absorbance was detected on a Multi-Detection Microplate Reader (BMG Labtech).

### Migration assay

Cell migration was evaluated using a wound-healing assay. Cells were seeded in a 6-well plate at 5 × 10^5^ cells per well and cultured for 24 h to ensure that the cells were almost confluent. The cells were starved with non-serum DMEM for 6 h and then scratched with a 10 μl pipette tip to create an artificial wound. After that, the cells were washed with serum-free DMEM and then incubated with or without miR-7-5p for 24 h. An Olympus IX70 inverted microscope (Shinjuku) was used to take images of the same field at 0 and 24 h. The cells that had migrated to the wound were quantified using Image-Pro Plus 6.0 software. The experiment was conducted in triplicate.

### Invasion assay

The invasion assay was conducted as previously described with some modifications [24]. Briefly, the upper chamber was pre-coated with Matrigel and 1 × 10^4^ cells suspended in serum-free DMEM were seeded in the upper chamber. At the same time, 600 μl of fresh normal DMEM was added to the lower chamber. After incubation for 24 h, the invading cells were fixed with 4% paraformaldehyde for 30 min and stained with 0.1% crystal violet. After that, the cells on the inner side of the chamber were removed with a cotton swab, and the invading cells on the lower surface were photographed with an Olympus IX70 inverted microscope. The invading cells were quantified using Image-Pro Plus 6.0 software. The experiment was conducted in triplicate.

### The transfection of miRNA mimic and vector

For miR-7-5p mimic transfection, adherent cells seeded in a 6-well plate (about 80–90% confluence) were transfected with 10 nM miR-7-5p mimic or NC mimic using Lipofectamine RNAiMAX transfection reagent (Invitrogen) following the manufacturer’s protocol. For the transfection of the pCMV3-NOVA2-GFPSpark and the pCMV3-C-GFPSpark vectors, Lipofectamine LTX & PLUS reagent was used according to the manufacturer’s instructions. After 6 h transfection, the cells were cultured for another 48 h and harvested for western blotting to determine the transfection efficiency.

### Dual-luciferase reporter gene assay

The dual-luciferase reporter gene assay were performed in line with the manufacturer’s protocol using the Dual Luciferase Assay System (Promega, cat. no. E1960). Cell lysates were used to determine luciferase activities with the dual luciferase reporter gene assay (Promega). Briefly, cells were transfected with pGL3-NOVA2–3′-UTR-WT or pGL3-NOVA2–3′-UTR-mut vectors that were detected with firefly luciferase once they were active. The cells were also transfected with pGL3 [hRluc/SV40] vectors that express Renilla luciferase to support the analysis of transfection efficiency. After a 24-h transfection, the cells were harvested and the luciferase signals were detected using a TECAN Infinite F500 platform with the Dual-Luciferase Reporter Assay System. The relative activity of the two luciferases were measured and calculated as ΔC_T_. The experiment was conducted at least three times.

### Western blotting assay

The western blotting assay was performed as previously described with some modification [25]. Briefly, after treatment with miRNA mimic for 24 h, the cells that were transfected with or without NOVA2 vectors were collected and lysed with RIPA buffer (Sigma-Aldrich). The protease and phosphatase inhibitors (Roche) were added to the RIPA buffer. Equivalent amounts of proteins were used for the western blotting assay. The quantitative data were analyzed with ImageJ software (NIH). Results are presented as respective ratios of β-actin.

### In vivo assay

A549 cells (1 × 10^7^) suspended in PBS were inoculated subcutaneously into the backs of BABL/c (nu/nu) male mice. When the tumor grew to about 200 mm^3^, the mice were randomly divided into the NC mimic group and miR-7-5p mimic group with five mice in each group. The mice were intravenously injected with NC mimic or miR-7-5p mimic every two days for 18 days. A slide caliper was used to measure the tumor volume with the formula: a × b^2^ × 0.5, where a refers to the longest diameter and b refers to the shortest. At the end of the experiment, the mice were scarified and the tumors were removed and frozen for further assay.

### Statistical analysis

All the data were analyzed with GraphPad Prism 5.0 (GraphPad Software) and the results are presented as the means ± standard error of the mean (SEM). The Pearson correlation coefficient was used for correlation tests between miR-7-5p and NOVA2. Differences were considered significant when *p* < 0.05.

## Results

### MiR-7-5p is downregulated in NSCLC tissues and cell lines

**Fig. 1:**
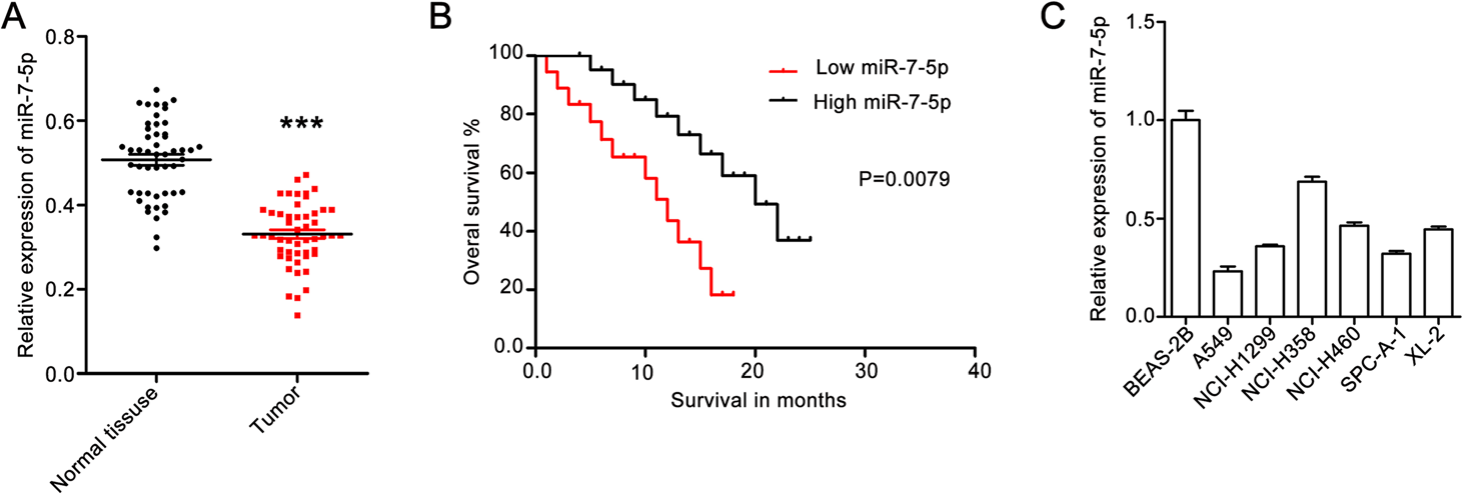
MiR-7-5p is downregulated in NSCLC tissues. **a** – The level of miR-7-5p in NSCLC tissues and adjacent non-tumor tissues. MiR-7-5p expression in 50 paired NSCLC tissues and adjacent non-tumor tissues was determined using qPCR. Quantitative data are presented as the means ± SEM. ***p < 0.001 compared with normal tissues. **b** – Kaplan-Meier curves for overall survival analysis as it correlates to miR-7-5p expression. **c** – MiR-7-5p expression in human lung epithelial BEAS-2B cells and the human NSCLC cell lines A549, NCI-H1299, NCI-H358, NCI-H460, SPCA-1 and XL-2. Quantitative data are presented as the means ± SEM. ***p < 0.001 compared with the control group

To clarify the significance of miR-7-5p in human NSCLC metastasis, the miR-7-5p level in 50 pairs of NSCLC tissues and matched para-carcinoma tissues were investigated using qPCR. The miR-7-5p levels were lower in NSCLC tissues than in paracarcinoma tissues (Fig. 1a).

Then the patients were divided into two groups according to their miR-7-5p expression and performed an overall survival rate analysis with Kaplan-Meier method. The results showed that the patients with low miR-7-5p expression (relative miR-7-5p expression lower than 0.5) had a poorer survival rate than those with high miR-7-5p expression (relative miR-7-5p expression greater than 0.5; Fig. 1b). This indicates that miR-7-5p plays a vital role in NSCLC.

In addition, we determined miR-7-5p expression in NSCLC cell lines (A549, NCI-H1299, NCI-H358, NCI-H460, SPCA-1 and XL-2) and in human normal epithelial BEAS-2B cells. The results showed that miR-7-5p expression in several NSCLC cell lines was higher than that in BEAS-2B (Fig. 1c). The expression of miR-7-5p was lower in A549 and SPCA-1 cells than that in cells of the other NSCLC cell lines. Based on these findings, miR-7-5p may suppress NSCLC tumorigenesis.

### MiR-7-5p inhibits migration and invasion abilities in A549 and SPCA-1 cells by downregulating EMT markers

We selected A549 and SPCA-1, which had the lowest miR-7-5p expression among the NSCLC cell lines, to validate the hypothesis that miR-7-5p may suppress NSCLC tumorigenesis. A549 and SPCA-1 cells were transfected with miR-7-5p mimic or NC mimic. The transfection efficiency was determined using qPCR. We then evaluated the effect of miR-7-5p.

**Fig. 2:**
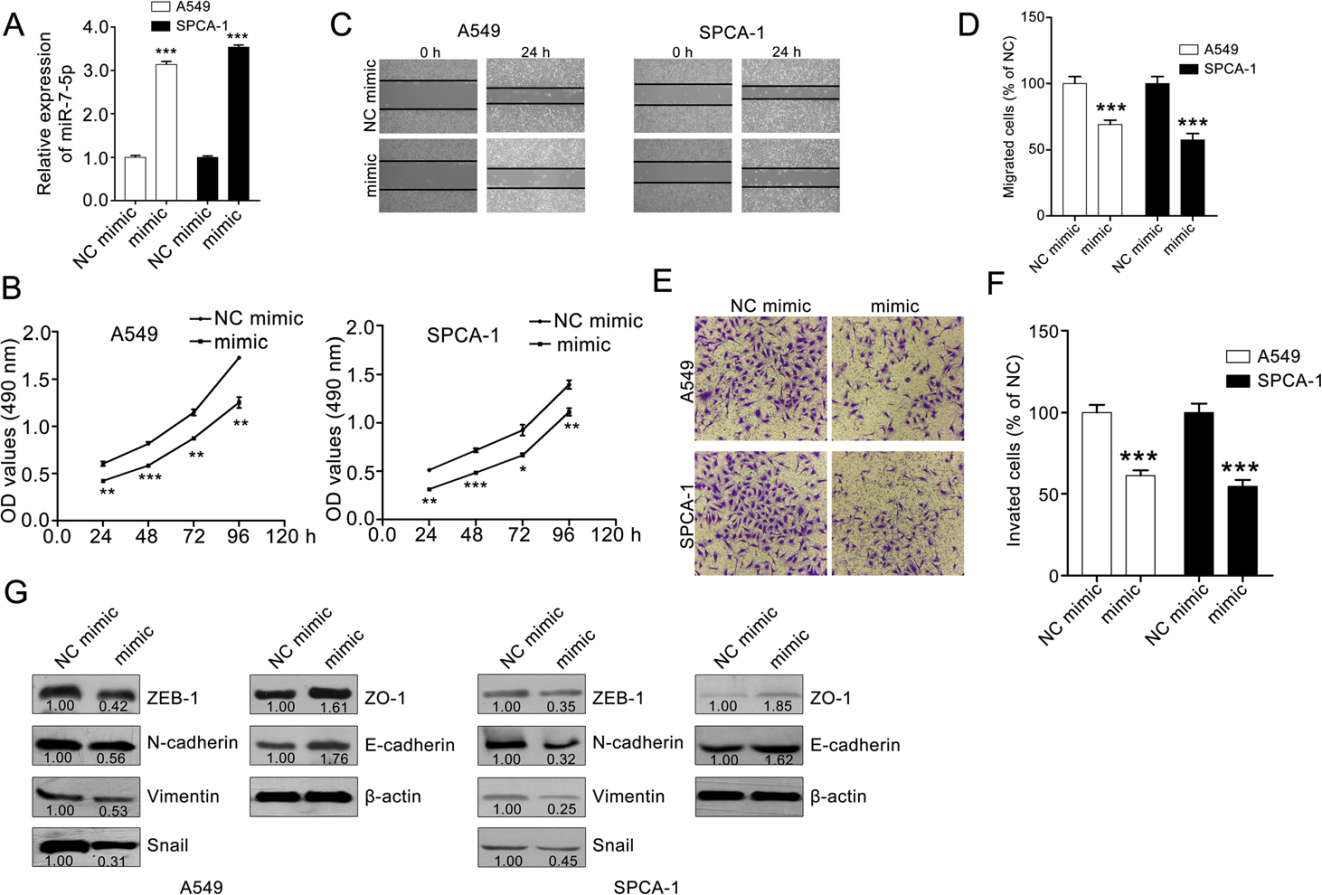
MiR-7-5p inhibits the proliferation, migration and invasion of A549 and SPCA-1 cells. **a** – qPCR assay confirming the transfection efficiency of the miR-7-5p mimic. **b** – MiR-7-5p suppressed the proliferation of A549 and SPCA-1 cells. Adherent A549 and SPCA-1 cells were cultured with or without miR-7-5p for 24, 48, 72 and 96 h, and the cell viability was then deteed using the MTT assay. **c** and **d** – MiR-7-5p inhibited the migration of A549 and SPCA-1 cells. Confluent cells were starved with serum-free medium and scratched with a pipette tip. After washing with PBS, the cells were cultured with or without miR-7-5p for 24 h. Representative images of the same field were photographed (100× magnification) at 0 h and 24 h. Representative images and quantitative data are shown in C and D, respectively. **e** and **f** – MiR-7-5p suppressed the invasion of A549 and SPCA-1 cells. A total of 2 × 10^4^ cells was seeded in the upper chamber of the transwell and treated with or without miR-7-5p for 24 h. After removing the cells on the inner wall of the upper chamber, the invading cells were photographed (100× magnification). Representative images and quantitative data are shown in E and F, respectively. The data were analyzed with GraphPad Prism 5.0. The data are presented as the means ± SEM, *n* = 3. ***p < 0.001 compared with the NC mimic group. **g** – MiR-7-5p downregulated EMT markers in A549 and SPCA-1 cells. β-actin was used as a loading control. The quantitative data were western blotting assays were measured with ImageJ software. Data are the ratios of EMT marker to β-actin

The results show that miR-7-5p inhibits the proliferation, migration and invasion abilities of A549 and SPCA-1cells. MiR-7-5p treatment effectively reduced the cell viability of A549 and SPCA-1 at 24, 48, 72 and 96 h (Fig. 2a). The effect of miR-7-5p on the migration abilities of A549 and SPCA-1 cells was measured using a wound-healing assay. The results show significantly fewer migrated cells in the miR-7-5p mimic group than in the NC mimic group (Fig. 2b and c). To further investigate whether miR-7-5p inhibits A549 and SPCA-1 cell invasion, a transwell invasion assay was conducted. The results show that the invasive capability of A549 and SPCA-1 cells was weakened after miR-7-5p treatment (Fig. 2d and e).

The expression of EMT markers in A549 and SPCA-1 cells were also determined using the western blotting assay. The results show that miR-7-5p treatment downregulated the level of ZEB1, N-cadherin, vimentin and snail, and upregulated the expression of epithelial markers, such as E-cadherin and ZO-1 (Fig. 2f) [26, 27]. Therefore, this study suggests that miR-7-5p inhibits the metastasis of A549 and SPCA-1 cells in vitro.

### MiR-7-5p directly targets NOVA2

**Fig. 3:**
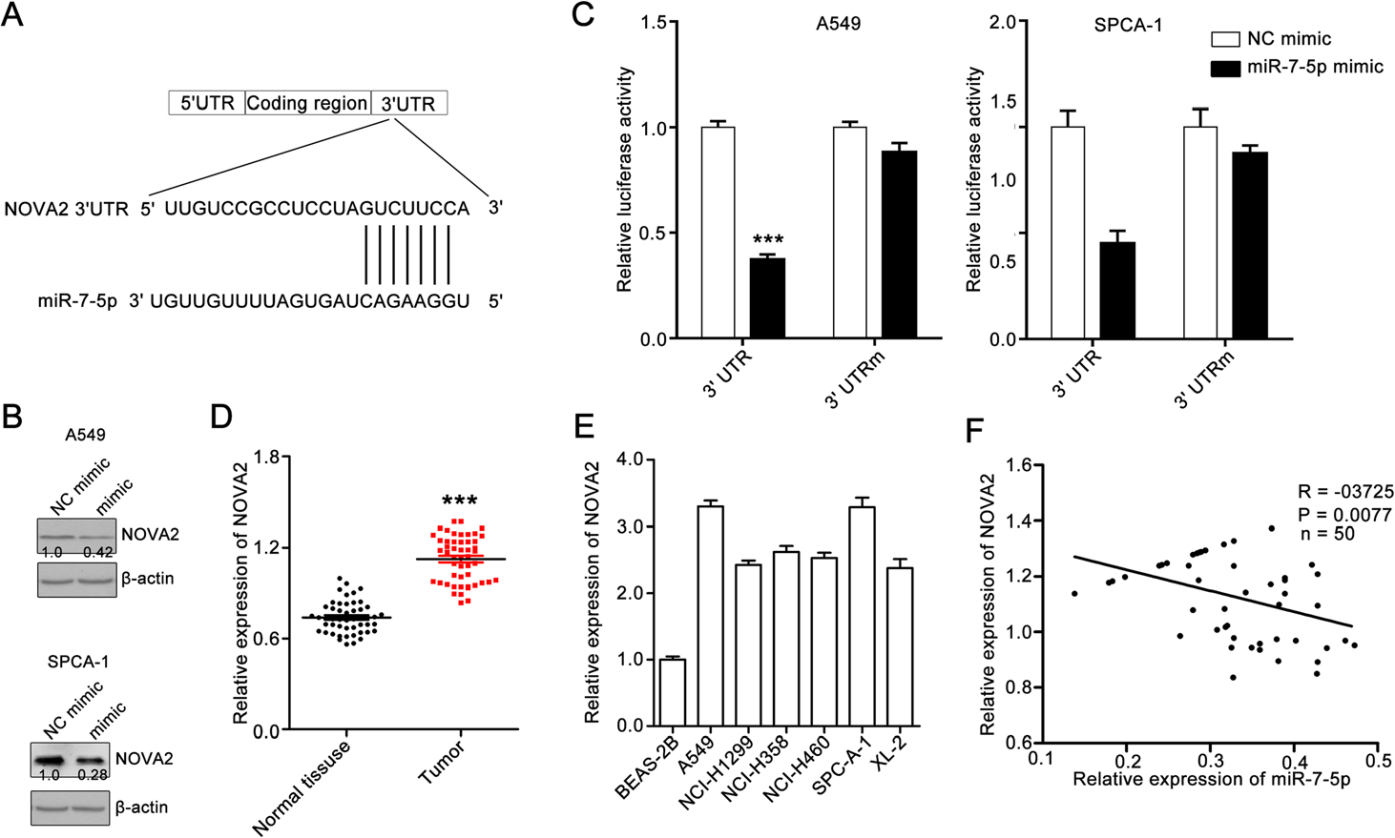
MiR-7-5p directly targets NOVA2 expression. **a** – The sequence of human miR-7-p and the predicted binding sites with miR-7-5p within the NOVA2 untranslated region (3′-UTR) are shown. **b** – MiR-7-5p treatment suppressed NOVA2 expression in A549 and SPCA-1 cells. The cells were cultured with or without miR-7-5p for 24 h, and then collected and used for western blotting assays to determine NOVA2 expression. β-actin was set as a loading control. The quantitative data from western blotting assays were measured with ImageJ software. Data are ratios of NOVA2 to β-actin. **c** – MiR-7-5p stimulation inhibited NOVA2 mRNA in A549 and SPCA-1 cells. A549 cells were co-transfected with luciferase plasmids containing the wild-type (WT) NOVA2 3′-UTR or mutant-type (Mut) NOVA2 3′-UTR. The cells were also treated with miR-7-5p at the same time. The cells were lysed to measure the relative luciferase activity. Quantitative data are presented as the means ± SEM, n = 3. ***p < 0.001 compared with the NC mimic group. **d** – NOVA2 expression in NSCLC tissues and adjacent non-tumor tissues was measured using qPCR. Quantitative data are presented as the means ± SEM. ***p < 0.001 compared with the NC mimic group. **e –** NOVA2 expression in a panel of human lung cell lines and human lung epithelial BEAS-2B cells. NOVA2 expression in BEAS-2B cells was set as 100%. Quantitative data are presented as the means ± SEM, n = 3. ***p < 0.001 compared with the BEAS-2B group. **f** – Analysis of the correlation between miR-7-5p and NOVA2 expression in tumors. NOVA2 expression was inversely correlated with miR-7-5p expression in NSCLC tissues. The miR-206 mRNA level was set as the X axes, and the TFR1 mRNA level was set as the Y axes. R stands for goodness of fit. The *p* value stands for slope significance

To determine how miR-7-5p dysregulation contributes to the migration and invasion of NSCLC cells, the potential targets of miR-7-5p were explored with the TargetScan tool. NOVA2 was identified as a potential target of miR-7-5p (Fig. 3a). We found that NOVA2 expression in A549 and SPCA-1 cells was downregulated following treatment with miR-7-5p (Fig. 3b), indicating that miR-7-5p may target NOVA2. To further confirm whether NOVA2 is a direct target of miR-7-5p, a full-length wild-type NOVA2 vector and mutant 3′-UTR of NOVA2 vector were constructed, and then were applied for a dual-luciferase reporter gene assay. The results show that the luciferase activity levels of pGL3-NOVA2-wt in A549 and SPCA-1 cells decreased obviously. However, miR-7-5p lost its inhibitory effect upon transfection of pGL3-NOVA2-mut containing the seed region mutation in cells (Fig. 3c).

In addition, the NOVA2 expression in NSCLC tissues and cell lines were determined. The results show that the NOVA2 expression is upregulated in NSCLC. NOVA2 expression in NSCLC tissues was significantly higher than that in paracarcinoma tissues (Fig. 3d). NOVA2 expression level was higher in NSCLC cell lines including A549, NCI-H1299, NCI-H358, NCI-H460, SPCA-1 and XL-2 than in BEAS-2B human lung epithelial cells (Fig. 3e). A significant inverse correlation between miR-7-5p and NOVA2 expression in NSCLC tissues were also observed. The patients with low miR-7-5p expression usually had high NOVA2 expression (Fig. 3f). Thus, it appears that miR-7-5p regulates NSCLC metastasis by directly targeting NOVA2.

### NOVA2 overexpression reverses miR-7-5p-mediated inhibitory effect on NSCLC cell metastasis

**Fig. 4:**
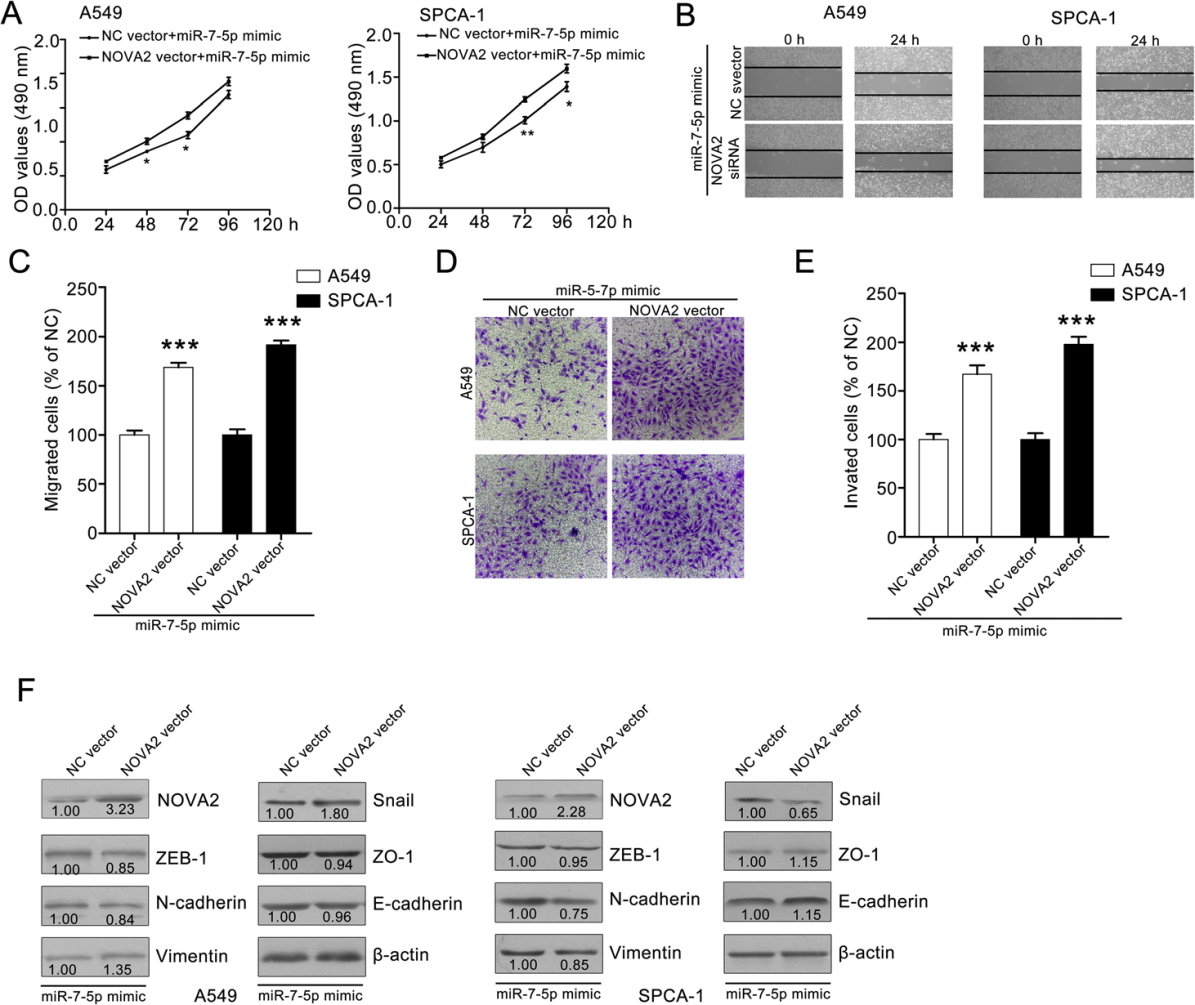
NOVA2 overexpression reverses the inhibitory effect of miR-7-5p on A549 and SPCA-1 cells. **a** – NOVA2 overexpression partly decreased the miR-7-5p-mediated inhibitory effect on the proliferation of A549 and SPCA-1 cells. **b** and **c** – NOVA2 expression weakened the miR-7-5p-induced inhibitory effect on the migration of A549 and SPCA-1 cells. A549 and SPCA-1 cells were transfected with NOVA2 vector. After 24 h, the transfected cells were used for wound-healing assays. Representative images and quantitative data are shown in B and C, respectively. **d** and **e** – NOVA2 overexpression attenuated the miR-7-5p-mediated effect on the invasion of A549 and SPCA-1 cells. Representative images and quantitative data are shown in D and E, respectively. Quantitative data are presented as the means ± SEM. ***p < 0.001 compared with NC vector group. **f** – NOVA2 overexpression blocked miR-7-5p-mediated downregulation of EMT markers. A549 and SPCA-1 cells were transfected with NOVA2 vector or NC vector and then treated with miR-7-5p for 24 h. After that, the cells were collected and used for western blotting assays. The quantitative data from western blotting assays were measured with ImageJ software. Data are ratios of respective EMT marker to β-actin

To further validate that miR-7-5p suppresses the migration and invasion abilities of NSCLC cells by targeting NOVA2, A549 and SPCA-1 cells were transfected with NOVA2 vector and evaluated the effect on proliferation, migration and invasion. The transfection efficiency were determined with a western blotting assay and found that NOVA2 vector transfection significantly increased NOVA2 expression in A549 and SPAC-1 cells. A549 and SPCA-1 cells that overexpressed NOVA2 were incubated with or without miR-7-5p and applied for the cell viability assay. The inhibitory effect of miR-7-5p on A549 and SPCA-1 cells partially decreased after NOVA2 overexpression (Fig. 4a). The miR-7-5p-mediated inhibitory effect on the horizontal migration of A549 and SPCA-1 cells was weakened after NOVA2 overexpression (Fig. 4b and c). The result also showed that NOVA2 overexpression reversed the miR-7-5p-mediated inhibitory effect on transwell migration and invasion abilities in A549 and SPCA-1 cells (Fig. 4d and e).

In addition, NOVA2 overexpression attenuated the miR-7-5p-mediated downregulation of vimentin, snail, slug, ZEB1, N-cadherin, E-cadherin and ZO-1 expression. The expression of vimentin, snail, slug, ZEB1, N-cadherin, E-cadherin and ZO-1 in NOVA2 overexpression cells was not significantly different for miR-7-5p mimic-treated cells and NC mimic treated cells. These data show that miR-7-5p negatively regulates the proliferation, migration and invasion abilities and EMT phenotype of A549 and SPCA-1 cells by targeting NOVA2.

### MiR-7-5p decreases tumor growth and metastasis in A549 xenografts

**Fig. 5:**
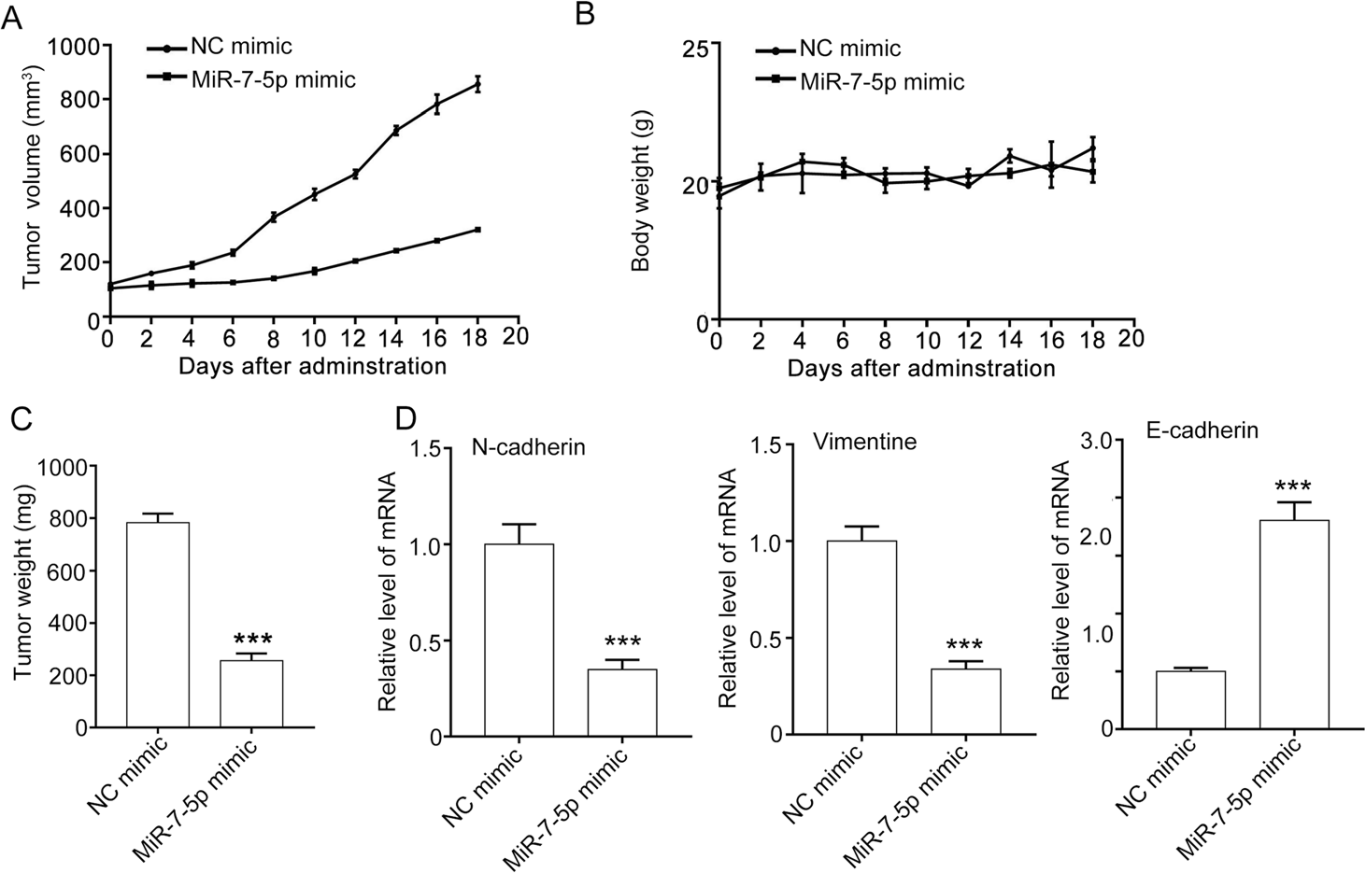
MiR-7-5p suppresses tumor growth and metastasis in vivo. **a** – miR-7-5p suppressed tumor growth, as measured by tumor volume. A549 cells (1 × 10^7^ cells per mouse) were subcutaneously injected into 5- to 6-week old mice. When the tumor had grown to about 100 mm^3^, the mice were injected intravenously with NC mimic or miR-7-5p mimic every two days for 18 days. **b** – MiR-7-5p had minimal effect on the body weight of the mice. **c** – MiR-7-5p inhibited the tumor growth, as measured by tumor weight. **d** – MiR-7-5p decreased the expression of EMT markers in A549 xenografts. At the end of the experiment, the mice were killed, and the tumors were removed and used for the qPCR assay to determine the mRNA level of N-cadherin, vimentin and E-cadherin. Quantitative data are presented as the means ± SEM. ***p < 0.001 compared with the NC mimic group

The in vivo effect of miR-7-5p mimic was evaluated using A549 xenografts. The results show that miR-7-5p significantly suppressed the tumor growth. The tumor volume in the NC mimic group increased from 120.30 ± 4.80 mm^3^ to 856.15 ± 41.52 mm^3^, and in the miR-7-5p mimic group from 111.6 ± 4.37 mm^3^ to 321.35 ± 11.78 mm^3^. The tumor weight in the miR-7-5p mimic group was 782.05 ± 31.40 mg, which was much lower than that in the NC mimic group (255.47 ± 24.90 mg). Furthermore, miR-7-5p mimic had minimal effect on the body weight of the mice (Fig. 5a–c).

In addition, miR-7-5p significantly suppressed the EMT in A549 xenografts, as indicated by the downregulation of N-cadherin and vimentin and upregulation of E-cadherin at the mRNA level in the tumor (Fig. 5d). These data suggest that miR-7-5p inhibits tumor growth and metastasis in A549 xenografts.

## Discussion

Several studies have shown that miR-7-5p contributes to the metastasis of gastric cancer and melanoma cells [17, 28]. However, the expression, biological function and molecular target(s) of miR-7-5p in NSCLC remain unclear.

NSCLC is one of the most common malignancies and its high worldwide mortality is a major concern [29]. Although considerable effort has been made to improve NSCLC therapy, the outcome remains poor. The invasiveness and metastasis of tumor cells is one of the most critical challenges hindering NSCLC therapy [29].

Many recent studies have suggested that miRNAs play a critical role in tumor metastasis through regulation of multiple oncogenes and tumor suppressor genes [11, 30, 31]. For example, miR-195 suppresses proliferation, migration, invasion, and tumorigenicity by targeting MYB in NSCLC [32]. Those authors also proposed that the miR-195/MYB axis has potential as a therapeutic target in NSCLC. MiR-193a-3p and miR-193a-5p suppress NSCLC metastasis by downregulating the ERBB4/PIK3R3/mTOR/S6K2 signaling pathway and overexpression of the two miRNAs blocks NSCLC metastasis [33]. MiR-638 levels decrease in NSCLC patients, and it function as a metastasis suppressor in NSCLC cell lines [20].

A previous study demonstrated that miR-7-5p plays a key role in suppressing tumor progression. MiR-7-5p inhibits the proliferation, migration and invasion abilities of multiple cancer types by targeting different genes [14]. For example, miR-7-5p suppresses proliferation and metastasis by regulating the PI3K/Akt signaling pathway in hepatocellular carcinoma and glioblastoma [34]. MiR-7-5p can also inhibit cell metastasis by targeting focal adhesion kinase (FAK) and Kruppel-like factor 4 (KLF4) in breast cancer [15, 35]. Recently, miR-7-5p has been shown to inhibit invasion and metastasis by downregulating epidermal growth factor receptor (EGFR) expression in gastric cancer cells [36, 37]. However, whether miR-7-5p can regulate NSCLC metastasis remains unclear.

This study assessed the expression and function of miR-7-5p in NSCLC. The results show that miR-7-5p is downregulated in NSCLC tissues and cell lines. Furthermore, we showed that it suppresses the proliferation, migration and invasion abilities of A549 and SPCA-1 cells by targeting NOVA2. MiR-7-5p can also suppress tumor growth and metastasis in A549 xenografts. The study indicates that targeting miR-7-5p may improve the survival rate of NSCLC patients thanks to its NOVA2 targeting-based disincentive role.

## Conclusion

The results show that miR-7-5p suppresses the proliferation, migration and invasion abilities of NSCLC cells by directly targeting NOVA2. This provides persuasive evidence for the vital role of miR-7-5p in NSCLC metastasis and indicates that miR-7-5p is a promising molecular target in NSCLC therapy.

## Data Availability

All data generated or analyzed during this study are included in this published article.

## Abbreviations

3′-UTR: 3′-untranslated region
EMT: Epithelial–mesenchymal transition
FBS: Fetal bovine serum
GBM: Glioblastoma multiforme
miRNA: microRNA
NOVA2: Neuro-oncological ventral antigen 2
NSCLC: Non-small cell lung cancer
